# Psychometric properties of the German version of the Self Image Scale (SIS-D) in a sample of cancer patients

**DOI:** 10.1371/journal.pone.0240619

**Published:** 2020-10-14

**Authors:** Jan Brederecke, Tanja Zimmermann

**Affiliations:** 1 Department of Psychosomatic Medicine and Psychotherapy, Hannover Medical School, Hannover, Germany; 2 Department of General and Interventional Cardiology, University Heart Center Hamburg, Hamburg, Germany; Medical University Innsbruck, AUSTRIA

## Abstract

**Background:**

A cancer disease can affect the satisfaction with the physical appearance, so that the standardized assessment of the body image is important in cancer patients. The German version of the Self-Image Scale is a self-report measure that uses two subscales to assess appearance satisfaction (self-acceptance) and perceptions of partners’ acceptance of their appearance (partner-acceptance). The present study aimed to validate the Self-Image Scale’s two-factor structure in a sample of cancer patients with a variety of different diagnoses to further increase its utility.

**Methods:**

Confirmatory factor analysis methods were used to examine the two-factor model in a sample of cancer patients (*N* = 278). Scale reliability and validity were then assessed through internal consistency measures and correlations with external criteria such as depressiveness, anxiety, distress, and relationship satisfaction.

**Results:**

The proposed factor structure was supported by the results and the internal consistencies were good with α = .83 for the self-acceptance scale and α = .88 for the partner-acceptance scale while additional correlations with external criteria were observed as expected.

**Conclusions:**

The results support the use of the German version of the Self-Image Scale in cancer patients in general. Future research directions include validation across further entities of cancer, the in-depth investigation of sex differences, and research in other diseases that might impact body image.

## Introduction

For long, cancer was treated as a purely physical disease and thus, successful treatment was mostly measured in survival rates and recurrence-free survival [1]. But as the numbers of patients and survivors continually grew throughout the past decades, the interest in the quality of their lives increased as well. Because of the body altering nature of the disease and many of its treatments, researchers and healthcare providers are more and more concerned with the effects of cancer and cancer treatment on patient’s and survivor’s body image as a part of their health-related quality of life [2]. The body image construct that is commonly referred to in the field of psycho-oncology generally includes a broad set of thoughts, feelings, and perceptions towards one’s body, and involves its functioning as well as its effects on the person’s quality of life [3–6].

Various studies have addressed body image and its different facets in the context of permanent body alterations like postoperative scars and amputations (e.g. [3, 7, 8]). Temporary changes in body image through side effects (e.g. hair loss, lymphedema, skin irritation) of nonoperative therapies like chemotherapy, radiotherapy or hormonal treatments have also been studied extensively (e.g. [9–11]). Nonetheless, the actual extent to which the body image in patients with cancer might be impaired in comparison with healthy people is not yet fully investigated. An extensive systematic review by Lehmann, Hagedoorn, and Tuinman [12] reported only 25 studies that compared cancer survivors with control groups from the general population. Additionally, the authors could not confirm that the body image of cancer survivors is decreased when compared to the general population as nearly half of the studies reported no significant difference between the control groups and the cancer populations, and three studies even found body image in the examined group of cancer survivors to be more positive [12]. Nevertheless, the reported studies used heterogeneous measures and assessed a range of different facets of body image [12]. Additionally, several measures were experimenter derived and methodological issues were common across the referenced studies which added up to the unclear results [12].

As most body image measures used in psycho-oncological research have been developed for use in specific cancer populations, especially female breast cancer patients, the comparison to other cancer populations and the general population is usually difficult [13]. Two of the more common measures were both developed with the *European Organization for Research and Treatment Center* (EORTC). While the EORTC’s breast cancer-specific module of the *Quality of Life Questionnaires Battery* (QLQ-BR23 [14]) was constructed exclusively for female breast cancer patients, the *Body Image Scale* (BIS [15]) was designed to work with any form of cancer while including the QLQ-BR23’s body image subscale. Both measures are well-validated [15], and especially the BIS has become a standard in body image assessment in clinical as well as research settings due to its brevity and comprehensive assessment of cancer patients’ body image. But since the majority of studies investigate body image only in the most prevalent cancer entities and almost exclusively in female cancer survivors, the knowledge beyond this group is still limited [2].

Nonetheless, a growing interest in research on male cancer patients’ and survivors’ body image indicates that men have body image difficulties that are associated with the cancer diagnosis and the side effects of its treatments, too. This can manifest in many different ways including changes to the sense of masculinity, the sexual self-concept, and sexual functioning [16–19].

Something that has often been omitted in the field of body image research in cancer patients is the relational aspects of body image in the context of intimate relationships. As was shown via dyadic experimental research in the general population, there is a significant social dimension to body image in that self-image can be influenced by intimate relationships, particularly in women [13]. Also, cancer and cancer treatments can negatively impact romantic relationships in multiple ways, e.g. by an impaired feeling of sexual attractiveness as well as diminished sexual communication, and functioning [20]. The perceptions of their partners' appraisals may be particularly important for female breast cancer patients’ adjustment as women’s perceptions of partners’ reactions to their appearance after surgery predict women’s sexual, marital as well as emotional adjustment [21].

Despite a small amount of available research, there is evidence for the body image of men to have a relational component regarding their intimate partner, too [22]. Different studies have shown a strong association between body image and relationship quality for both women and men [23], and that psychological processes and perceived relationship quality in intimate relationships can influence body image, dieting behavior [24], and sexual satisfaction in women as well as men [23].

Comparative research on the intimate partner relational component of body image in cancer patients versus in the general population lacks measures that assess the perceived partner responses to appearance. Thus, measures for body image that are designed and validated to work with multiple groups of cancer patients, that can be used across the sexes, and that were used to generate normative data for comparison with the general population are needed to answer the question of body image impairment in cancer patients more broadly.

One instrument that has been validated in the general population and that takes the relational aspects of body image into account is the German version of the *Self-Image Scale* (SIS-D). The SIS-D is the German version of the Self Image Scale (SIS) that was originally developed to assess body image adjustment in Australian women with cancer, especially breast and gynecological cancer [25]. The version translated into German was initially used to study individual and dyadic variables relevant for female cancer patient’s body image in German breast cancer patients and their partners [26]. The SIS-D’s factor structure was then validated in a representative sample of the general German population [27]. For the aforementioned study, all femininity specific items were also presented in a masculinity specific version to include men (e.g., “I think my partner sees me as a woman/man”). The SIS-D contains eleven items that are assigned to two scales: The *self-acceptance* scale (six items) assesses a persons’ sense of femininity/masculinity as well as a persons’ acceptance of his or her appearance (e.g., “I like the way I look in my clothes”) while the *partner-acceptance* scale (five items) assesses a persons’ perceptions of their partner’s responses to, and acceptance of their appearance (e.g., “I think my partner sees me as a woman/man”). The SIS-D’s assessment refers to the past week and the individual items are rated using a five-point Likert-type scale (1 = *strongly disagree*; 5 = *strongly agree*). As two of its items are negatively formulated, they have to be recoded afterward. Scale scores range from 6 to 30 for the self-acceptance scale while the partner-acceptance scale score ranges from 5 to 25 and higher scores indicate greater acceptance.

The SIS-D’s psychometric properties in the sample of the German general population were good with internal consistencies (Cronbach’s α [28]) of .82 for the self-acceptance scale and .89 for the partner-acceptance scale [27]. This was in line with former findings in female breast cancer patients with α ranging from .83 to .92 for self-acceptance and from .82 to .91 for partner-acceptance [29]. The SIS-D has also been demonstrated to be measurement invariant across age groups and the sexes, and is thus fit for the use in men as well as women of different age [27].

### Study objectives

The present study ties in directly with former research that validated the SIS-D’s two-factor structure in the German general population [27], and aims to validate the SIS-D’s two-factor structure in a sample of male and female cancer patients across a range of different diagnoses. Additionally, the reliability as well as the validity of the SIS-D regarding depressive symptoms, anxiety, cancer-specific psychological distress, and relationship satisfaction will be considered.

## Method

### Data sampling

The data in this cross-sectional study were acquired through responses of 285 cancer patients collected in a regional hospital in Germany. Participants filled out paper-pencil versions of the survey. Participants were asked to answer the German versions of the *Patient Health Questionnaire* (PHQ-9 [30]) and the *General Anxiety Disorder Screener* (GAD-7 [31]) as measures of depression and anxiety along with the SIS-D items. They also answered the German versions of the *Questionnaire on Stress in Cancer Patients revised version* (QSC-R23 [32]) and the *Quality of Marriage Index* (QMI [33]) as measures of distress and relationship quality, respectively.

Additionally, all participants provided basic demographic information and accepted written informed consent prior to their participation in the study. The study has been approved by the ethics committee of the Technische Universität Braunschweig, Germany (B-2016-05).

### Sample characteristics

In order to be able to provide meaningful information regarding the partner-acceptance scale of the SIS-D, all participants had to be in a relationship at the time of the survey. Additionally, seven (~2.5%) persons that took part were excluded afterwards because they did not respond to any of the questions. This resulted in a final sample size of *N* = 278. Participants were diagnosed with a range of different kinds of cancers that were aggregated into the following groups: *breast and gynecological cancer*, *prostate and testicle cancer*, *visceral cancer* (e.g. lung, liver, and colorectal cancer) and *other cancers*. Detailed demographic characteristics of the sample are presented in Table 1. Men and women did not differ in a statistically significant way regarding age, time since diagnosis, years of education, and employment status. But, as could be expected because of a range of sex specific diagnoses included in the sample, the sexes differed regarding the type of cancer (Table 1). The amount of missing values in the present sample was 2.9% overall.

**Table 1:** Demographic characteristics of the sample by sex.

	Men	Women	Total	*p*^a^
	(*n* = 142; 51.5%^b^*)	(*n* = 134; 48.6%)	(*N* = 278)	
**Age** (*M*, *SD*)**	57.10 (10.42)	55.68 (8.33)	56.39 (9.45)	.211
**Months since Diagnosis** (*M*, *SD*)**	19.29 (23.02)	17.83 (18.27)	18.58 (20.77)	.564
**Years of Education** (*n*, %)***				.168
≤ 9 years	79 (55.6%)	57 (42.5%)	137 (49.3%)	
10 years	33 (23.2%)	39 (29.1%)	73 (26.3%)	
>10 years	26 (18.3%)	31 (23.1%)	57 (20.5%)	
other	4 (2.8%)	7 (5.2%)	11 (4.0%)	
**Employment status** (n, %)***				.138
Employed	135 (95.1%)	119 (90.2%)	256 (92.8%)	
Unemployed	7 (4.9%)	10 (7.6%)	17 (6.2%)	
Other	-	3 (2.3%)	3 (1.1%)	
**Type of Cancer** (*n*, %)***				**< .001**
Breast & Gynaecological	1 (0.1%)	81 (61.8%)	84 (31.3%)	
Prostate & Testicle	37 (27.4%)	-	37 (13.8%)	
Visceral	37 (27.4%)	23 (17.6%)	60 (22.4%)	
Other	60 (44.4%)	27 (20.6%)	87 (32.5%)	

*Notes*.

^a^*p* of the Sex Comparison.

^b^All percentages were calculated from valid cases only and sums of more than 100% in a category result due to rounding to one decimal place.

*Two participants did not specify their sex.

**t-Test.

***Fisher’s exact test.

### Additional measures

#### The German version of the Patient Health Questionnaire (PHQ-9)

Symptoms of depression were assessed using the German version of the PHQ-9 [30]. The nine items of the PHQ-9 correspond to symptoms of major depressive disorder in the Diagnostic and Statistical Manual of Mental Disorders [DSM- IV-TR; 34]. Items are rated on a four-point Likert-type- scale ranging from 0 (*not at all*) to 3 (*nearly every day*). The PHQ-9 total score ranges from 0 to 27, and higher results indicate higher depressiveness. A cut-off of ≥10 indicates a moderate depression. In the present study, the internal consistency was good with α = .83.

#### The German version of the General Anxiety Disorder Questionnaire (GAD-7)

Symptoms of anxiety were assessed using the GAD-7 [31]. The seven items of the GAD-7 correspond to symptoms of general anxiety disorder in the DSM-IV-TR [34]. Items are rated on a four-point Likert-type- scale ranging from 0 (*not at all*) to 3 (*nearly every day*). The GAD-7 total score ranges from 0 to 21 and higher scores indicate higher levels of anxiety and scores ≥10 indicate a general anxiety disorder. The internal consistency was good in the present study (α = .87).

### The German version of the Questionnaire on Stress in Cancer Patients Revised Version (QSC-R23)

Cancer-specific psychological distress was assessed with the QSC-R23 [32]. The QSC-R23 measures psychosocial stress in cancer patients through 23 items. The items describe potential stressors in different areas. The items have to be answered twice: Whether they apply to the participant, and if so, to what extent. The items are thus answered on Likert-style scales ranging from 0 (*the problem does not apply to me*) to 5 (*the problem applies to me and is a very big problem*). The items can be grouped to five scales: *psychosomatic complaints*, *fears*, *information deficits*, *everyday life restrictions* and *social strains*. In this study, only the total sum score was used. Higher scores indicate more distress and a cut-off of ≥ 34 indicates significant cancer related stress [32]. The internal consistency of the QSC-R23 in this study was good with α = .92.

### The German version of the Quality of Marriage Index (QMI)

Relationship satisfaction was assessed through use of the German version of the QMI [33]. Participants are asked to evaluate their agreement with statements regarding their relationship (dis-)satisfaction. Participants answer the first five items on a 7-point Likert-type scale that ranges from 1 (*strongly disagree*) to 7 (*strongly agree*). The sixth item asks respondents to rate their overall level of happiness on a 10-point scale ranging from 1 (*extremely low*) to 10 (*extremely high*). The sum-score (range:6–45) of the items was used in the present study and higher scores indicate higher relationship satisfaction. A cut-off of 34 divides those satisfied from those not satisfied with their relationship [33]. The reported internal consistency of the QMI in this study was high with α = .95.

### Statistical analysis

The SIS-D’s two factor structure that was originally proposed by Scott, Halford, and Ward [25] and validated in the general German population by Brederecke et al. [27] was tested using confirmatory factor analysis (CFA) methods. All CFA related calculations were done using the *R* [35] packages *lavaan* [36], *semTools* [37] and *mice* [38]. In order to account for the 4.3% (range: 2.2%-9.4%) missing values in SIS-D items alone, multiple imputation methods included in the mice package were used in factor analysis related computations. All other calculations were done using valid case analysis. The CFAs were calculated for *m* = 20 multiply imputed datasets and the results were pooled according to *Rubin’s rules* [39]. A weighted least squares estimator that uses diagonally weighted least squares as well as mean and variance adjusted test statistics (WLSMV) was utilized to estimate parameters of the CFA models as the SIS is measured on a categorical scale [40].

Model fit was then assessed using the following fit indices: χ^2^ test statistic for absolute fit, comparative fit index (CFI; [41]) for fit relative to a null model, Root Mean Square Error of Approximation and 90% Confidence Interval (RMSEA; [42]) and Standardized Root Mean Square Residual (SRMR; [43]) for overall fit. The χ2 test statistic is sensitive to large sample sizes and thus tends to reject models in larger samples [44]. Therefore, it was only included for completeness only. According to Hu and Bentler [45], good model fit can generally be assumed when CFI and/or TLI is higher than 0.95 (>0.90 is acceptable), SRMR is smaller than 0.08 and RMSEA is smaller than 0.06 (<0.09 is acceptable). Additionally, the final CFA model was again calculated using *listwise-deletion* for cases with missing values. Results were then compared to the model that was based on the multiply imputed datasets in order to see if bias was introduced.

Internal consistency of the SIS-D subscales was assessed by calculating the Cronbach’s α coefficient for both subscales of the SIS-D. Additionally, the SIS-D scale scores were correlated with the PHQ-9, GAD-7, QSC-R23 and QMI scores as well as age as measures of validity. Point biserial correlations and effect sizes (*Hedges’ g* [46]) were used to determine the relationship of self-acceptance and partner-acceptance with sex.

All statistical analyses were performed in R. All tests were based on a significance level of .05 (if not stated otherwise).

## Results

### Confirmatory factor analysis

The first CFA model showed an unacceptable model fit with χ^2^ = 223.795, *df* = 43, *p* < .001, RMSEA = .12 (CI = .11-.14), CFI = .89, SRMR = .07. In order to see if the model fit could be improved, error covariances of variables with overlaps in content were freed, as similar content is known to have negative impact on model fit when corresponding error covariances are not freed [47]. Namely, error covariances of items 1 and 2 were freed to correlate with each other as were the error covariances of items 4 and 9, 5 and 7, 3 and 10, and 8 and 11. This was done analogous to the original validation study [27].

**Fig 1:**
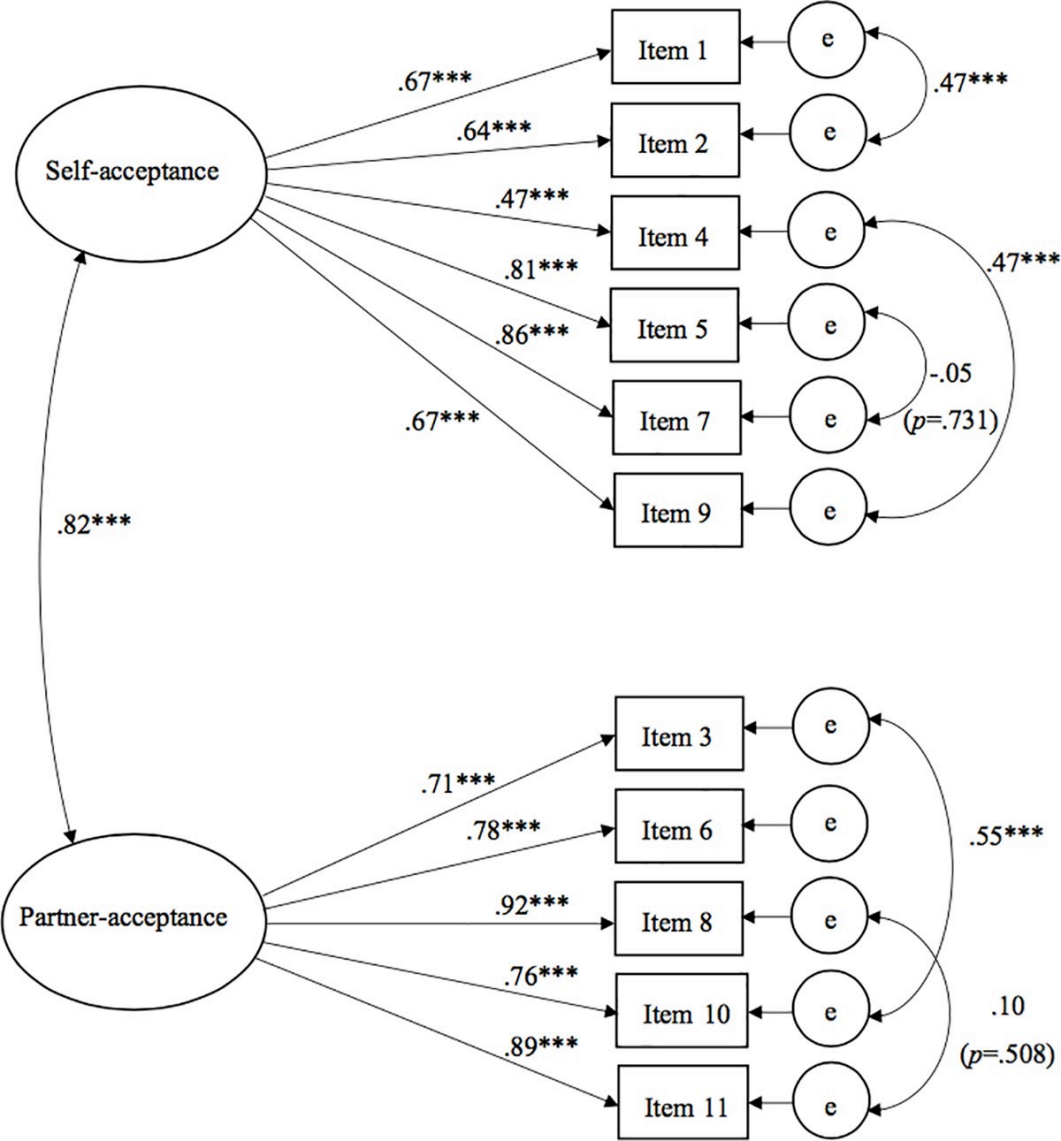
Standardized factor loadings, factor correlations and error covariances of the SIS-D. ***p < .001. Correlation of Self-acceptance and Partner-acceptance *r* = .82***. The values next to the straight arrows represent the standardized factor loadings that can be interpreted as correlations of the respective factor and item. The values next to the curved arrows are the freed error covariances of the corresponding items.

A scaled χ^2^-difference test [48] showed the refit model to differ in a statistically significant way from the initial model, χ^2^(5) = 117.275, *p* < .001. Additionally, it then showed a good model fit with χ^2^ = 71.241, *df* = 38, *p* = .001, CFI = .98, RMSEA = .06 (CI = .04-.08), and SRMR = .04. A high correlation between the factors (*r* = .82, *p* < .001) indicated that the constructs self-acceptance and partner-acceptance are considerably overlapping in content but still below a threshold (*r* = .85) that was proposed by Cohen et al. [49]. Table 2 shows the SIS-D’s items and their respective factor loadings in the final model (Fig 1). The model that was fit using complete cases only (*n* = 240) showed an equally good model fit with χ^2^ = 76.686, *df* = 38, *p* < .001, CFI = .99, RMSEA = .07 (CI = .04-.09), and SRMR = .04.

**Table 2:** Items and factor loadings of the final SIS-D two-factor model.

No.		Original item (German version)	Unstandardized loading (standardized)^a^	*M*(*SD*)	Corrected item-total correlation^b^
Self-acceptance				
1	I like the way I look undressed. (Ich mag mein Aussehen, wenn ich unbekleidet/nackt bin.)	**1**^c^ (.67)	2.93 (1.11)	0.66
2	I like the way I look in my clothes. (Ich mag es, wie ich in meiner Kleidung aussehe.)	**.95** (.64)	3.47 (0.98)	0.58
4	I avoid looking at myself. (Ich vermeide es, mich selbst anzusehen.)	**.71** (.47)	4.11 (1.07)	0.46
5	I feel attractive. (Ich finde mich attraktiv.)	**1.22** (.81)	3.06 (0.93)	0.64
7	I feel good about myself. (Ich finde mich gut.)	**1.29** (.86)	3.26 (0.94)	0.64
9	I dislike my appearance. (Ich mag meine Erscheinung nicht.)	**1.00** (.67)	3.87 (1.04)	0.60
Partner-acceptance				
3	I think my partner sees me as a woman/man. (Ich denke, dass mein Partner/meine Partnerin mich als Frau/Mann sieht.)	**1**^c^ (.71)	3.97 (1.11)	0.64
6	I think my partner enjoys sexual intimacy with me. (Ich denke, dass mein Partner/meine Partnerin sexuelle Intimität mit mir genießt.)	**1.1** (.78)	3.53 (1.17)	0.66
8	I think my partner finds me attractive. (Ich denke, dass mein Partner/meine Partnerin mich attraktiv findet.)	**1.29** (.92)	3.61 (1.00)	0.77
10	I think my partner sees me as feminine/masculine. (Ich denke, dass mein Partner/meine Partnerin mich weiblich/männlich findet.)	**1.07** (.76)	3.84 (1.05)	0.70
11	I think my partner finds me sexy. (Ich denke, dass mein Partner/meine Partnerin mich sexy findet.)	**1.24** (.89)	3.48 (0.98)	0.78

*Notes*.

^a^All factor loadings were statistically significant (*p* < .001).

^b^Correlation of the item with the scale without the item.

^c^One factor loading per factor was set to 1 to scale the factor [36].

### Reliability

Both scales of the SIS-D showed good internal consistencies with α = .83 for self-acceptance and α = .88 for partner-acceptance. This suggests good internal validity of the SIS-D subscales in this sample of cancer patients.

### Validity

Correlations of the SIS-D scales with the PHQ-9 and GAD-7 were *r* = -.42 (*p* < .001) and *r* = -.40 (*p* < .001) for the self-acceptance subscale and *r* = -.23 (*p* < .001) and *r* = -.23 (*p* < .001) for the partner-acceptance subscale. Self-acceptance additionally correlated positively with the QMI (*r* = .31, *p* < .001) and negatively with the QSC-R23 total scale (*r* = -.44, *p* < .001). The partner-acceptance scale also correlated positively with the QMI (*r* = .38, *p* < .001) and negatively with the QSC-R23 total scale (*r* = -.33, *p* < .001). The correlations with age were *r* = -.03 (*p* = .634) for the self-acceptance and *r =* -.16 (*p* = .009) for the partner-acceptance scale. Point biserial correlations of the SIS-D subscales with sex (0 = female; 1 = male) were *r* = .20 (*p* = .001, *g* = .400) for self-acceptance and *r* = -.05 (*p* = .405, *g* = .103) for partner-acceptance.

## Discussion

The present study examined the SIS-D’s two factor structure via CFA methods in a sample of cancer patients. In contrast to previous studies regarding the SIS, the sample consisted of both men and women and a wide range of cancer diagnoses were included. Additionally, the internal consistency of the SIS-D’s scales and their validity were examined.

The results of the factor analyses implicate that the SIS-D’s two-factor solution that has previously been validated only in female breast cancer patients as well as the German general population (fit indices of the two-factor model in the German general population: RMSEA = .11 (CI = .10-.11), CFI = .98, and SRMR = .04 [27]) is also valid in a wider range of cancer diagnoses. In order to arrive at a well-fitting CFA model, minor modifications to the model were conducted. Thus, multiple correlations between errors of reverse worded and similarly worded items were included in the model as was done in the original validation study before [27]. The identified model then confirmed that the two scales self-acceptance and partner-acceptance can be described as two distinct constructs even though the two factors are highly correlated [49]. The high factor correlation is not surprising as satisfaction with one's own body and the perceived satisfaction of a partner with one' s body are likely related and at least partly explained by dyadic interactions (e.g. [50]). The differentiation of the scales is essential for the practical value of the SIS-D. Especially when used as a screening tool, the partner-acceptance scale’s results can be used to explore topics like relationship quality and sexuality, subjects that are often vital for patients but skipped or avoided by professionals in psycho-oncologic care [51]. The high internal consistencies of the SIS-D’s subscales indicate good reliability of the scales and are consistent with previous results. Regarding validity, the correlations with depressive symptoms and symptoms of anxiety were all observed as could be expected from the literature as well as former findings regarding the SIS-D [27, 52]. Additional correlations with measures of relationship satisfaction and distress were also in line with the available literature (e.g. [16, 53]). In a previous study [26] in which the SIS was used in couples with breast cancer, relationship satisfaction emerged as an important predictor of partner acceptance, showing that women who have cancer and live in a happy relationship worry less about their partner's reactions to their physical appearance. Other findings also show that a happy relationship also seems to have an influence on self-image [54]. The correlations with age partly differed from the general trend that could be deduced from the SIS-D’s validation in the German population [27]. While the previous study implied that there is a general tendency towards lower self-acceptance with increasing age, no significant correlation of age and self-acceptance could be reported in the present study. The results on age and self-acceptance are inconsistent. In a study of women with breast cancer, older women were more dissatisfied with their body image than younger ones [26]. Other studies do not show any correlation between age and body image [55]. Nonetheless, the significant negative correlation of age and partner-acceptance is in line with former findings from the validation study as well as the broader research on body image in older adults [56, 57]. The significant correlation of the SIS-D’s self-acceptance scale was observed as expected from the literature [58, 59], with a tendency towards lower self-acceptance for women. In line with the validation study, no significant correlation of sex and the partner-acceptance facet of body image was observed in the present study.

### Limitations

First and foremost, the sample size in the present study limits the scope of the results. In addition, the sample included a relevant proportion of female breast cancer patients that have already been subject to former validation studies of the original SIS that was breast cancer specific. Moreover, the present findings are based on a German sample. Thus, inferences to the English version of the SIS are not possible.

### Strengths

As this is the first study to examine the factorial structure of the SIS-D in a population of male and female cancer patients with a wide range of cancer diagnoses, the results contribute significantly to the field of psycho-oncology as well as body image research generally. Only a few studies have examined body image in men with cancer and the current findings thus contribute to the field. Moreover, the results implicate that the SIS-D can be used in a wider range of cancer patients and the present study thereby increases the SIS-D’s applicability.

### Clinical implications

In line with former findings, the results of the present study suggest that the SIS-D is applicable in general psycho-oncological practice to assess changes in self- and partner-acceptance over time in response to either medical treatment or psychological intervention. The SIS-D’s brevity helps to effectively incorporate it in psycho-oncologic care and its partner-acceptance results might help clinicians to explore sensitive topics like intimate relationship quality or sexuality.

## Conclusion

The results of the present study support the assumed factor structure the SIS-D’s factor structure is not only valid in women with breast-cancer and the German general population but also in cancer patients across the sexes and with different kinds of cancers. The SIS-D seems thus fit for clinical use in cancer patients in general. Future research is necessary to further evaluate the SIS-D’s psychometric properties in different kinds of cancers and to generate normative data in other languages to enable meaningful cross-country comparisons. Especially the comparison of cancer patients with the general population and the comparison of male and female cancer patients should be focused in upcoming studies. Methodological refinement could mean using new approaches like using the Expanded format instead of the usual Likert-scale in order to improve the SIS-D’s factor structure [60]. Additionally, research in samples that are exclusively male, in populations with other chronic medical conditions like transplantation, heart disease, diabetes, and multiple sclerosis might also be included in the future.

## Supporting information

S1 Dataset This SPSS dataset was used for all calculations in the present study. (SAV)
